# Evaluation of *Helicobacter pylori* OipA protein as a vaccine candidate and propolis as an adjuvant in C57BL/6 mice

**DOI:** 10.22038/ijbms.2021.56232.12579

**Published:** 2021-09

**Authors:** Hengameh Soudi, Tahereh Falsafi, Mohaddeseh Mahboubi, Sara Gharavi

**Affiliations:** 1 Rajaie Cardiovascular Medical and Research Center, Iran University of Medical Sciences, Tehran, Iran; 2 Microbiology department, Faculty of Biological Sciences, Alzahra University, Tehran, Iran; 3 Medicinal Plants Research Department, Research and Development, TabibDaru Pharmaceutical Company, Kashan, Iran; 4 Biotechnology Department, Faculty of Biological Sciences, Alzahra University, Tehran, Iran

**Keywords:** Adjuvant, Helicobacter pylori, IFN-γ, IL-4, OipA, Propolis, Vaccine

## Abstract

**Objective(s):**

Outer inflammatory protein A (OipA) is an essential adhesin of *Helicobacter pylori*. We aimed to evaluate the effects of a recombinant OipA in the induction of crucial cytokines as a vaccine candidate and propolis as an adjuvant in C57BL/6 mice.

**Materials and Methods:**

C57BL/6 mice were divided into nine groups according to the disposition of antigen and adjuvant and route of administration: subcutaneous (sc) or gavage. The administrated recombinant purified OipA and propolis concentrations were 10 μg/ml and 40 μg/ml, respectively. After vaccination, we measured expression levels of IFN-γ and IL-4 cytokine genes in the spleen cells of mice by real-time PCR.

**Results:**

All results were contrasted with the negative sample. By sc injection, the expression of INF-γ was increased 3.5 and 2.9-fold for OipA and OipA plus propolis, respectively. By gavage 4.4 and 11-fold increase was found for OipA and OipA plus propolis, respectively. The administration of propolis by gavage showed more increase than Sc injection concerning the production of INF-γ. The 11-fold increase for injection of OipA plus propolis by gavage was comparable OipA plus Freund’s adjuvant injected subcutaneously. This result suggested an excellent immunological response toward OipA concerning the production of INF-γ in mice. In all cases there were no notable IL-4 production increases.

**Conclusion:**

The results confirm the efficiency of OipA in induction of IFN-γ production, and thereby the cellular immune response. Propolis could be a suitable adjuvant.

## Introduction

*Helicobacter pylori* (*H. pylori*) colonize the gastric epithelium, are related with peptic ulcer and considered a gastric carcinoma and gastric mucosa-related lymphoid tissue lymphoma (MALT) risk factor (1). The customary treatments for *H. pylori* infections are the proton pump inhibitors with combinations of two distinct antibiotics, including amoxicillin, metronidazole, clarithromycin, and tetracycline. Nonetheless, due to the presence of safe strains in numerous geographic locales, effective eradication has failed, and the repeat of infection contributes to the long-lasting infectivity of *H. pylori* (2). Alternative therapeutic or prophylactic strategies are needed to manage the complications of *H. pylori* infections. In this approach, developing a vaccine requires appropriate bacterial antigens combined with an effective adjuvant. The proper administration route may also be critical for its effectiveness. During the past decades, different measures such as co-administration of immunomodulatory molecules have been taken, improving the efficacy of vaccines. Chemokines are one of the primary key elements in controlling immune responses and lead to the migration of immune cells in both innate and adaptive responses. These chemokines are immunomodulatory molecules and have been studied as the adjuvant in both cancer and infectious vaccination studies.

The results of previous studies demonstrated that human gastric mucosa infected by *H. pylori* carried an increased level of proinflammatory cytokines, such as IL-8, IL-1, TNF-α, and IL-6 (2, 3). A group of cytokines named IFN-α, IL-12, and IL-18 involved in recruiting Th1-directed cellular immune responses is also overexpressed (4, 5).

In any case, despite the production of various types of cytokines, the host’s immune response is commonly deficient to eradicate infection. Also, researchers observed the possible association of the genetic polymorphisms, causing a higher production of the same inflammatory cytokines with an expanded danger of gastric malignancy (2, 6, 7). Previous investigations also indicated that among the inflammatory cytokines, IFN-γ in directing the appropriate Th1 immune response to *H. pylori* infection has a considerable role, in addition, a dual role of this cytokine, which is involved in the induction of pre-neoplastic changes in the gastric mucosa, has been reported in the trial infection models (8). The consequences of this investigation have shown that IFN-γ produced by CD4+ CD25+ effector cells are critical for *H. pylori* infection control, and they can incite pre-neoplastic modifications in the gastric mucosa. Such a change is ascribed to the degree of the IFN-γ response (8). Generally prophylactic and therapeutic vaccination procedures have been set up dependent on the determination of antigens obtained from *H*. *pylori* infection pathogenesis agents (9, 10). The primary observation of *H. pylori* urease has demonstrated that it cannot inhibit urease activity in animal models (11). 

Further investigations are performed on immunizations of mice with intact urease protein leading to secretion of two types of antibodies (MAbs); the first is the conformational epitope-explicit neutralizer against the S3 protein and the linear epitope-explicit immune response against the L2 protein (11-13). 

A study (14) compared the efficacy of subcutaneous and intranasal forms of epitope-based vaccines. The two forms of epitope-based vaccines exhibited a notable decrease in the bacterial load separate from the humoral immunity (14). However, priming of Th1-related immune responses, as well as a reduction in bacterial burden, carried in animals receiving the subcutaneous form of epitope-based vaccines in immunized animals, using cholera toxin B subunit (CTB) as a complete vaccine, led to acceptable immune responses in animal models (13, 15-18). Studies have demonstrated that this is safe when used as a vaccine element in humans (19-21).

The significance of outer inflammatory protein A (OipA), belonging to *H. pylori*, which stimulates IL-8 production, a proinflammatory cytokine, has been addressed by the literature (22). Th1 effector cells, through the creation of interferon-gamma (IFN-γ), as a critical cytokine, along with tumor necrosis factor (TNF)-α and -β mediate protection against H. pylori, has contributed to the infection (3, 23). While OipA can trigger the synthesis of IL-8 (2, 22), it may also be involved in the trigger of cytokines such as IL-1, IL-17, and TNF-α (4). 

OipA is a 34 kDa protein obtained from a functional *oipA* gene (5). The corresponding cloned protein may be like that of the protein proposed by Yamaoka and colleagues (24).

Even though OipA has a significant role in the host protection against *H. pylori* infection (25); obtaining the optimum results depends on selecting a proper and potent adjuvant to induce an efficient immune response against this antigen. To date, many adjuvants are examined, and most industrial vaccines still depend on utilization of aluminum salts or oil emulsions. Also, oil adjuvants are not at this point utilized for people because of their unfavorable impacts (6). The employment of natural adjuvants may be promising for the vaccination process, of which propolis shows remarkable adjuvant properties when utilized in animal models (26). Propolis or “bee glue,” is a resinous compound gathered by honeybees from the developed blossoms. It is a characteristic material delivered by bumblebees from various plants (27, 28). Propolis is chemically complex and composed of more than 300 different substances depending on the geographical regions (28, 29). Nevertheless, it has different biological and pharmacological features reported, exhibiting immunostimulatory and immunomodulatory properties (5, 25, 30-31). 

The purpose of this examination is to assess the immunogenic effect of a purified recombinant protein, OipA, as an antigen, and propolis as an adjuvant, in a murine model

## Materials and Methods

***Characteristics of the recombinant protein OipA***

*Identification of recombinant OipA Protein*

The recombinant OipA protein, expressed by the *oipA* gene, was taken from the *H. pylori* strain disengaged from a person with gastritis (25). After induction of expression of the recombinant plasmid in optimum conditions, OipA was purified by Ni-NTA affinity chromatography (Takara, Japan) using a hybrid method of denaturation and on-column re-solubilization. The cell pellet from the microbial culture was briefly collected, washed, and dissolved in a binding buffer solution (100 mmol/l NaH_2_PO_4_, 10 mmol/l Tris-HCl, and 8 mol/l urea; pH=8). Following the removal of the insoluble materials by centrifugation, batch purification method was performed by Ni-NTA agarose. The mixture was washed by denaturing wash buffer (100 mmol/LNaH_2_PO_4_, 10 mmol/l Tris-HCl, and 8 mol/l urea; pH= 6.3) followed by rinsing with denaturing wash buffer plus 8 M urea (pH= 5.9). The resins were washed with different wash buffers (50 mmol/l NaH_2_PO_4_, 300 mmol/l NaCl, 5% ethanol, pH 8) in descending concentrations of urea (8, 6, 4, 2, 1, and 0 mmol/l). Finally, the elution buffer (250 mmol/l imidazoles, 50 mmol/l NaH_2_PO_4_, 250 mmol/l NaCl, pH 8) was used to elute the recombinant OipA protein from the Ni-NTA agarose. purified fractions were analyzed by SDS-PAGE then pooled and dialyzed. The purified protein was detected by western blot analysis using an anti-His-tag monoclonal antibody (Sigma Aldrich, Germany). Alternatively, according to the previously reported protocol, this recombinant protein was identified using a rabbit monoclonal antibody against OipA (25). The purified protein was used as an antigen for immunization of a murine model of *H. pylori*. After the recombinant protein purification, lipopolysaccharide (LPS) was also removed from our last preparations by Polymyxin B sulfate (20 μg/ml) to suppress the stimulating and toxic effects of LPS in animals.

***Propolis preparation and its chemical analysis***

We employed Iranian propolis (Sepahan ASAL Company, Isfahan, Iran) as a natural adjuvant. To set up the adjuvant, propolis was ground and blended in with ethanol (70%), then stirred for 24 hr. The acquired concentrates were dried by evaporating the solvent at 50 ^°^C under vacuum. 

***Chemical analysis of propolis by GC and GC-MS***

For analyzing the chemical compounds in the extracts, 1 mg of the extracts was dissolved in 100% ethanol and analyzed by GC and GC-MS. GC and GC-MS analyses were done on an HP 6890 GC system coupled with a 5973-network mass selective detector with a capillary column of HP-5MS (30 mm×0.25 mm, film thickness 0.25 μm). The oven temperature was started at 60 ^°^C, held for 1 min, then increased to 245 ^°^C at a 3 ^°^C/min rate, and then held for 10 min. Helium gas was used as carrier gas at a flow rate of 1.5 ml/min. The detector and injector temperatures were 250 and 230 ^°^C, respectively. Retention indices (RI) were calculated for all components using a homologous series of n-alkanes at the same GC conditions. The compounds of the extracts were identified by comparing their RI values and mass spectral fragmentation with those in the stored Wiley 7n.1 mass computer library (32). The quantification of significant constituents of oils was assessed by the area normalization method (33).

To obtain the total phenolic contents of propolis, 10 mg of propolis was dissolved in 10 ml of ethanol. Then, 0.2 ml of the obtained solution was mixed with 3 ml of water and 0.25 ml of Folin-Ciocalteu reagent (10%) by agitation. After 3 min, 0.75 ml of 20 % (w/v) sodium carbonate was added, mixed well, and after adjusting to 5 ml, kept at room temperature for 1 hr. The total phenolic contents were measured by a standard curve generated with gallic acid at a wavelength of 760 nm. The results were expressed as percent (%) of the dried extract in equivalents of gallic acid.

The total flavonoid contents were determined by the aluminum chloride colorimetric method. To this aim, 0.5 ml of propolis extract was mixed with 0.1 ml of aluminum chloride (10%), 0.1 ml of potassium acetate (1 M), and 5 ml of distillate water. The absorbance was measured at a wavelength of 415 nm after 30 min, and the total flavonoid contents were calculated using a standard curve based on the concentration of quercetin. The results were expressed as percent (%) of the dried extracts (34).

***H. pylori strain preparation as a positive control***

The *H. pylori* strain, B19, with the *cagA/vacAs1m2* genotype was isolated from a patient and chosen for injecting into the mice as a positive control (25). According to our previous study, this strain could be colonized in the murine stomach.

The 3-day new culture of the *H. pylori* strain was gained on brucella agar (Merck; Germany) plus 7% sheep blood and necessary antibiotics.

***Mice immunization and procedures***

All experimental procedures were conducted according to the UK Animals Scientific Procedures Act of 1986 (86/609/EEC). The study was confirmed by the Animal Ethics Committees of AL Zahra University. The number of groups and the animals per group were determined by the ‘Resource Equation’ method. This approach is used for animal research, where the sample size calculation is required, but it is not possible to presume the standard deviation. It helps to set an acceptable range of error degrees of freedom in analyzing variance (ANOVA). This approach is used to give the possibility to presume the standard deviation for a smaller size of animals. It is intended to examine any changes in different groups and examine the hypothesis as the primary objective (35).

Pathogen-free five-week-old female C57BL/6 mice (Razi Institute; Karaj, Iran) housed in a clean environment with open access to food and water at a temperature of 21±2 ^°^C, 55±5% humidity, and 12:12 hr light/dark cycle. 

For mice immunization, the recombinant OipA protein and propolis proportions were selected based on the results obtained from our previous study conducted on human macrophage cell culture. The concentrations chosen for antigen and adjuvant yielded a huge amount of INF-γ (36). Our previous research showed optimum concentrations of the antigen and adjuvant to induce better production of INF-γ as a critical cytokine of the cellular immune response (36).

 Nine mice groups (5 in each group) were prepared, according to the nature of the antigen and/or adjuvant as well as the administration route: subcutaneous (sc) or gavage (Table 1). The amount of the administrated recombinant OipA and propolis was 10 μg/ml, and 40 μg/ml, respectively, in 200 microliters of experimental vaccines containing 130 microliters of OipA protein plus adjuvant at 30% of antigen volume. Mice receiving Freund’s as adjuvant received complete Freund’s adjuvant at the first injection and incomplete Freund’s adjuvants at the second and third injections (10 and 20 days later). Mice selected as positive control received three consecutive days with 0.2 ml of alive *H. pylori* 19B strain (1×10^8^ CFU/ml). We sacrificed all mice on the 10th day of the last booster dose injection, and their internal organs were removed to be analyzed.

***Evaluation of cytokine production and bacterial colonization***

Finally, mice were anesthetized by peritoneal infusion of 1.43 mg/kg diazepam (Khemidaru, Iran) and 13 mg/kg ketamine 10% (Alfasan, Woerden, the Netherlands) (25). Then, at that point, the stomach pit of mice was opened, and their spleens were gathered and weighed for RNA extraction and measurement of IFN-γ and IL-4. In the group receiving *H. pylori* as an antigen, the stomach was removed for determination of colonized *H. pylori* and checked of urease activity. For determination of colonized bacteria, gastric tissue was homogenized in Brucella broth mixed with 5% FCS, then cultured on the brucella agar medium and incubated for 5 days (37 ^°^C, microaerobic conditions). Colony recognition was performed after incubation. Urease action in the homogenized gastric tissue was performed according to the previously described method (37).

***Determination of IFN-γ and IL-4 expression by real-time PCR***

The expression levels of IFN-γ and IL-4 were measured by real-time PCR. The experimental procedures of gene expression analysis are presented below.

*Primer’s design*

The Primer 3 software was utilized to design specific primers, and the BLAST tool was applied to verify the specificity of the designed primers against whole gene-bank databases (Table 2). The primers were synthesized (Sinaclon, Iran). The primer efficiency in the real-time PCR reactions was evaluated using the Linreg PCR software.

*RNA extraction*

The total RNA was extracted from 25-50 mg of the spleen tissue using an RNeasy mini column according to the manufacturer’s protocol (Pars-tous biotechnology, Iran). The optical density of samples was measured at 260/280 ratios using a spectrophotometer to determine the quantity and purity of the extracted RNA.

*First-strand cDNA synthesis*

The extracted RNA was used to synthesize cDNA using the First-Aid Reverse Transcription Kit (Fermentas). In brief, 5-10 μg of the extracted RNA, 1 μl of oligo dT, 1 μl of the random hexamer, and 15-20 pmoles of sequence-specific primers were adjusted to 14 μl by DEPC water. The resulting composition was heated (65 ^°^C for 5 min), then cooled on ice and mixed rapidly. Then, 4 μl of RT 5 x buffer, containing 10 Mm dNTPs, 1 μl DTT, and RevertUP™ II Reverse Transcriptase were added to each microtube. It was then heated to 50 ^°^C for 60 min and incubated at 95 ^°^C for 5 min to deactivate the reverse transcriptase enzyme (terminal steps were performed at the thermocycler apparatus).

The prepared cDNA was then stored in a freezer and used as a template for quantitative PCR reactions. PCR primer sequences used for the amplification of the cytokine genes are shown in Table 1. 

*Real-time quantitative PCR*

The real-time PCR reactions were conducted to analyze the expression levels of IFN-γ, IL-4, and b-actin. In this section, the synthesized cDNA, and selected primers were used for q real-time PCR. The reaction conditions were as follows 95 ^°^C for 15 min, 95 ^°^C for 30 sec, 62 ^°^C for 30 sec , 72 ^°^C for 30 sec in 40 cycles, and 1 cycle at 72 ^°^C for the 30 sec for the final extension. All experiments were done in triplicate. The β-actin gene was used as the reference gene to determine an arbitrary normalized value for each gene. 

***Statistical analysis***

The statistical analysis of the obtained data was performed by The REST software version 9. The data were analyzed by ANOVA, followed by Tukey’s *post hoc* test. The level of the statistical significance was set at *P*<0.05.

## Results

***Characterization of recombinant OipA protein after purification***

**Figure 1.**
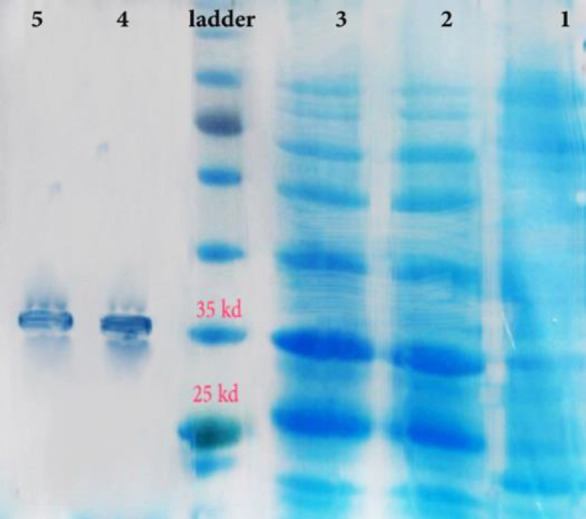
The extracted and purified recombinant OipA protein was from the bacterium. Protein display in 12.5% SDS-PAGE gel, first to the fifth row from right: 1- uninduced, 2 and 3- induced by IPTG, protein leader, 4 and 5- purified protein (36)

**Figure 2:**
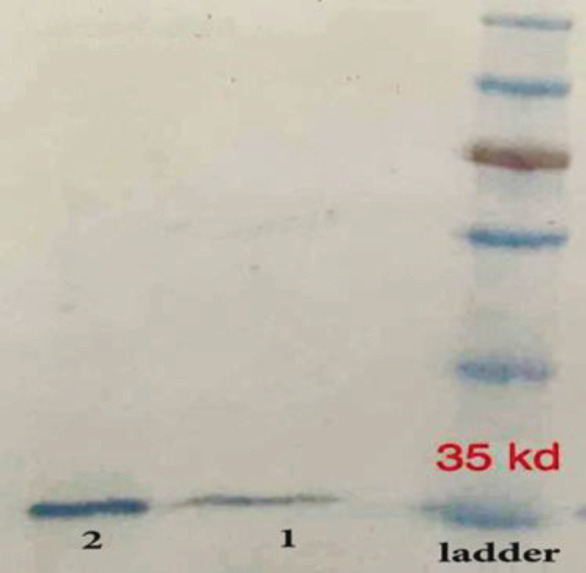
Proof of the presence of OipA protein and its identification by western blotting. From right: Ladder, 1 and 2: samples of the extracted protein (36)

The *oipA* gene was sequenced and contained 924 bp. The molecular weight of the purified recombinant OipA protein was about 33-35 kDa (Biomatic, Canada). The sequence similarity of the *oipA* gene with other recognized *oipA* genes was 92-96%. The most similarity was seen in the Mexican oipA clone (10). The optimum concentration of IPTG to achieve maximum production of recombinant OipA was 1 mmol/l. The recombinant OipA protein was identified by the western blot analysis using an anti-His-tag monoclonal antibody to detect a 34 kDa protein (Figures 1 and 2). 

For immunization of the mice, the concentrations of the recombinant OipA protein and propolis were 40 μg/ml and 10 μg/ml, respectively.

***Chemical compositions of propolis***

The chemical compositions of propolis employed in this work showed 18 compounds in the extracts that consisted of 99.9% of the total extract composition. These compounds included caffeic acid (16.2%), urea, N-benzyl-N’-methyl-N-(p-tolyl) (13.6%), 3-chloroisocoumarin (12.5%), and hydroxy-5-methoxyflavan (11.5%). The chemical compositions of propolis were determined by GC and GC-MS analyses and are depicted in Table 3.

***Assessment of H. pylori colonization in positive control mice group***

After separation of gastric tissue and culturing on an appropriate culture plate, smooth bacterial colonies were observed, showing that the stomach of mice was colonized well by this strain. Their identification was performed by the gram staining method and standard biochemical tests. 

***Evaluation of IFN-γ and IL-4 secretions in splenocytes of immunized mice***

**Figure 3:**
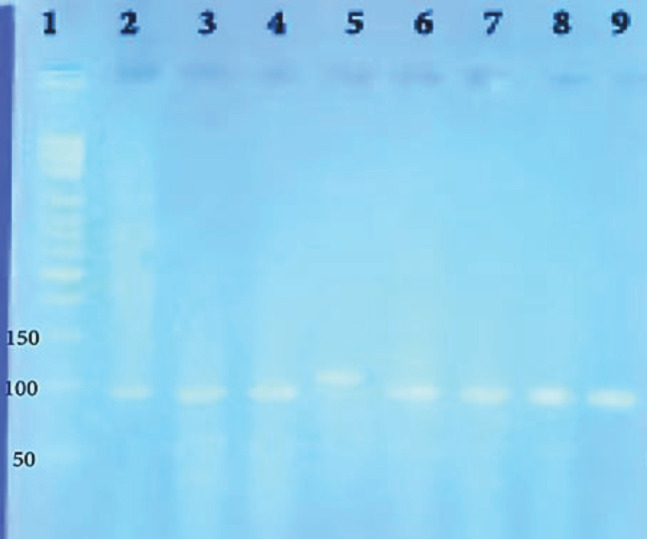
Evaluation of gene products: Lines 2-4; IFN-γ, Lane 5; Beta-actin, Lanes 6-9; IL-4

In this experiment, the expression levels of IFN-γ and IL-4 genes were determined by real-time qPCR and normalized against expression of the β-actin gene during each reaction. The efficiency of primers was assessed using the Linreg PCR software, and the REST software calculated the relative gene expression. To ensure the presence of the reaction product, the melting curve was calculated for all wells by the device. For each reaction, the product of PCR was subjected to electrophoresis (Figure 3). The results indicated that in comparison with the negative control, in all groups, the expression of IFN-γ was significantly increased, whereas the level of IL-4 was not altered or even decreased (Table 3).

**Figure 4:**
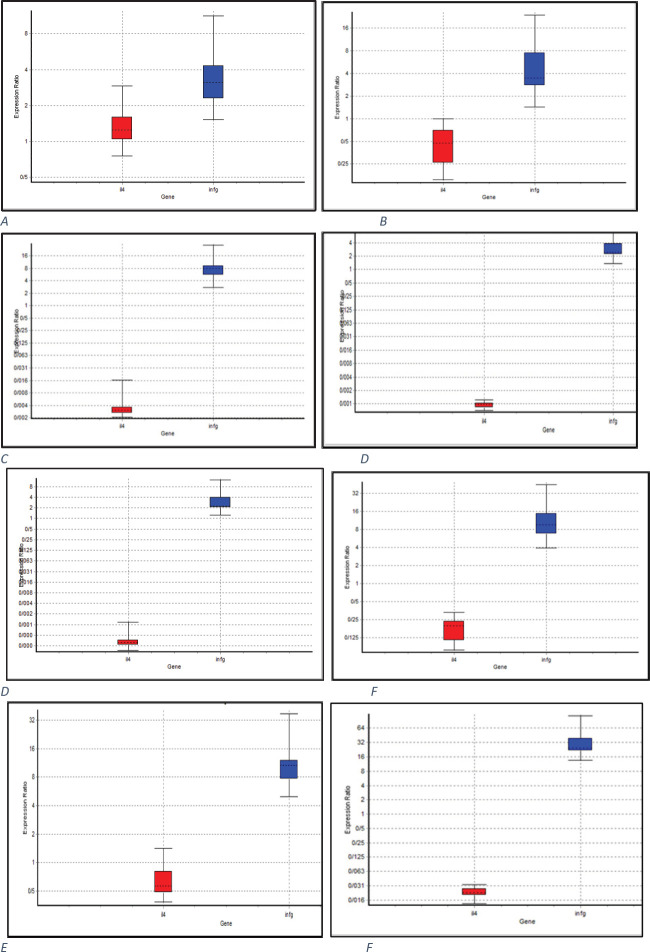
Relative expression report of IFN-γ and IL-4 in the spleen cells of mice in different treated groups in comparison of the negative group (mice treated by PBS), subcutaneously (sc) and via Gavage (gv). Boxes address the interquartile range or the center half of perceptions. The dotted line addresses the median gene expression. Whiskers represent the base and most observations (report produced by REST 2009 V2.0.13 © Copyright: 2009) (C) QIAGEN GmbH All rights reserved. A-group 1(sc: PBS+OipA); B-group 2 (gv: PBS+OipA); C- group 3(sc: propolis+PBS); D-group 4 (gv: propolis+PBS); E-group 5 (sc: OipA+propolis); F-group 6 (gv:OipA+propolis); G-group 7 (gv: Freund’s adjuvant+OipA); H-group 8 (positive control: *Helicobacter pylori*)

**Figure 5:**
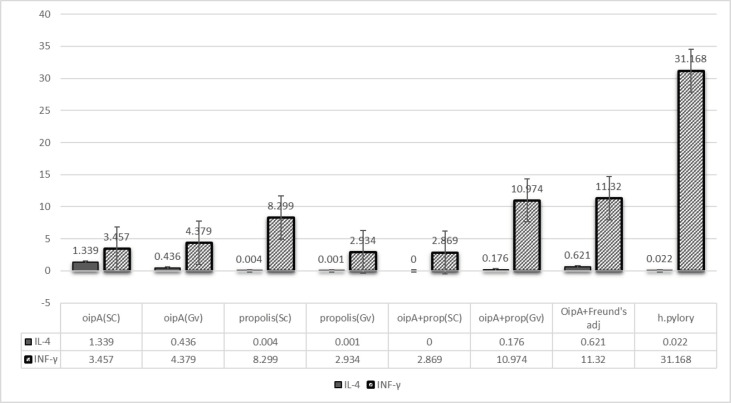
Results of different treatment protocols (relative expression of IL-4 and IFN- γ) in experimental groups compared with the negative control group (group treated only with PBS)

The relative expression of IFN-γ in mice receiving OipA by subcutaneous injection and oral routes demonstrated a significant difference (Figure 4, 5). The results indicated that in all experimental groups compared with the control, the expression of IFN-γ was markedly increased; however, the expression of IL-4 was not altered or even reduced. As shown in related figures (Figure 4), the expression of IFN-γ in group 1 treated with the subcutaneous form of the recombinant oipA protein was increased by almost (≈)3.5 folds; however, it was nearly increased by ≈ 4.4 folds in mice treated with the oral administration of OipA (Figures 4A, B). Such an increment was higher in mice treated with propolis plus the recombinant OipA protein. In mice treated with oral administration of OipA plus propolis, the expression of IFN-γ was higher (11 folds) than in mice treated with the subcutaneous form of propolis plus the recombinant OipA protein (Figures 4E, F).

Administration of propolis alone via the subcutaneous form showed similar results with OipA plus propolis, indicating a 2.9-fold increase compared with the negative control (Figure 4C). However, the amount of IFN-γ was significantly increased by 8.3 folds in mice receiving propolis alone via oral administration (Figure 4D). This data showed a stunning differentiation in the expression of IFN-γ between the mice receiving propolis by the oral route and those receiving via the subcutaneous route; it was an important observation. 

Mice treated with OipA plus Freund’s adjuvant showed an 11-fold increase (Figure 4G) in the expression of IFN-γ when compared with the control group. This result suggested an excellent immunological response toward OipA concerning the production of INF-γ in mice. In the case of the positive control group infected with *H. pylori*, a 31-fold increase in the expression of IFN-γ compared with the negative control group was observed (Figure 4H). 

**Table 1:** Characteristics of the different treated mice groups

Groupe	Administrated antigen and/or adjuvant	Administration route
1	OipA + PBS	Sc
2	OipA+ PBS	Gv
3	propolis	Sc
4	propolis	Gv
5	OipA + propolis	Sc
6	OipA + propolis	Gv
7	OipA + Freund's adjuvant	Sc
8	*H. pylori*	Gv
9	Negative control (PBS)	Gv

Sc: Subcutaneous; Gv: Gavage

**Table 2:** Physiochemical characteristics of the designed primers

Product length bp	TM (°C)	3՛-5՛	Primer sequences
78	60.08 60.50	ATCCTGCTCTTCTTTCTCGAATGT GCCGATGATCTCTCTCAAGTGATT	*il-4-F* *il-4-R*
101	61.39 61.53	TGTCCACCTTCCAGCAGATGT AGCTCAGTAACAGTCCGCCTAG	*Beta-actin F* *beta-actin R*
71	61.35 60.13	ACAATGAACGCTACACACTGCAT TGGCAGTAACAGCCAGAAACA	*ifn-γ -F* *ifn-γ -R*

**Table 3:** Chemical composition of propolis

Compound	RI	%
**Benzenemethanol, 3,5-dimethyl-**	1391	2. 4
**β-eudesmol **	1504	1.5
**α-eudesmol **	1508	1.5
**rosifoliol**	1627	1.1
**n-Hexadecanoic acid**	1795	2. 9
**hexadecanoic acid, ethyl ester**	1809	0.7
**(trans)-2-nonadecene**	1915	3.6
**oleic acid, 2-hydroxyethyl ester**	1928	1.7
**benzeneethanamine, N-[(4-hydroxy) hydrocinnamoyl]**	2045	7.3
**caffeic acid phenyl ethyl ester**	2077	16.2
**3-chloroisocoumarin**	2104	12.5
**7-hydroxy-5-methoxyflavan**	2134	11.5
**3,4,4A,4B,5,6-hexahydro-3,6-dioxo-9,10-diethylphenanthrene**	2163	6.2
**n-(4-methoxy-2-benzothiazolyl) benzamide**	2189	2.6
** 2-nitro-p-cresol**	2203	4.2
**Urea, N-benzyl-N'-methyl-N-(p-tolyl)-**	2237	13.6
**chrysin**	2259	4.1
**benzoic acid, 4-(phenylazoxy)-, ethyl ester**	2294	6.3

RI: Retention index; (%) = Percent of the peak area

## Discussion

The regulatory role of T helper cells (Th1 and Th2) in controlling and leading the immune system response against *H. pylori* infections is partially understood. However, it may be accepted that the most effective immune system response to overcome the infection caused by *H. pylori* is the cellular immune system. The feature of this system is an increase in IFN-γ and a decrease in IL-4, which subsequently results in a cascade of other effective cytokines (38). Following the initiation of infection, the earliest stages of host defense against inflammation begin with the tight attachment of bacteria to the gastric mucosa. Hence, the control of bacterial infection at this early stage seems to be an appropriate way to prevent further steps, which might be more severe. The virulence factors involved in this step could be suitable candidates for the development of vaccines.

OipA is one of the outer membrane proteins of *H. pylori* among the most effective adhesins of these bacteria, which plays a role in the attachment of bacteria to the gastric mucosa. It appears that this protein is expressed in virulent strains of *H. pylori* and stimulates an inflammatory response in the host immune system, leading to overexpression of IL-8.

It has been shown that the functional gene *oipA* was cloned, and its expression profile was examined (5). Another similar study demonstrated the production of anti-OipA IgA antibodies in C57BL/6 murine models (25). The current study aimed to investigate the efficiency of this protein in improvement of an effective vaccine. At first, we performed an *in vitro* experiment to analyze the purified recombinant OipA protein expression by the western blot analysis using an anti-His-tag antibody. The product exhibited a protein band at a molecular weight of 34 kDa. The western blot was also conducted with a rabbit monoclonal antibody against OipA (data not shown), which showed similar results with that of the anti-His-tag antibody. 

To assess the proper immunogenicity of the recombinant OipA protein to develop a vaccine and the efficiency of propolis, as a natural adjuvant, in the modulation of immune responses, these two substances were analyzed in our study. Propolis was selected for its safety as a natural adjuvant (3). 

Along with other *H. pylori* antigens, much effort has been made to evaluate the role of OipA as a protective vaccine in the prevention of *H. pylori* infection (2, 4). 

Although evolution of cellular or humoral immune responses relies upon a broad variety of cytokines secreted by some cells, such as CD4+ (Th1 and Th2) or CD8+ T cells, IFN-γ, produced by Th1 cells, is a crucial cytokine in directing the cellular immune response (28).

We have recently determined the role of OipA in the induction of IFN-γ and IL-4 in human macrophages (36). We observed that both IL-4 and IFN-γ were remarkably increased. However, such vital cytokine induction was dependent on the dose of antigens, since in higher concentrations of antigens, the expression of IL-4 was completely suppressed and the expression of IFN-γ reached the baseline. Despite a dose-dependent increase in the expression of both IFN-γ and IL-4, the defined concentration to induce the expression of IFN-γ was much higher than that of IL-4. In human macrophages, the optimum concentration of propolis resulted in the increased secretion of IFN-γ. Such an increment was 4 times higher than that of OipA.

C57BL/6 mice were used to evaluate the immunogenicity of the organism as described. We attempted to use the specified formulas in grouping mice and the number of mice in groups to save the consumption of mice and use fewer animals, which is also statistically acceptable. To evaluate the immunogenicity of OipA recombinant protein, we used the effective concentrations obtained in the *in vitro* cell culture method in our previous study. To compare the immunogenicity of propolis, OipA protein was used from another common adjuvant called complete Freund’s adjuvant, and the results were evaluated. Consumption of Freund’s adjuvant helped to evaluate the immunogenic potential of our antigen, which is the recombinant OipA protein, along with a conventional and proven adjuvant. Thus, if propolis did not show a suitable adjuvant effect, our studies were able to estimate the immunogenicity of the protein regardless of propolis, and on the other hand, the adjuvant potential of propolis was compared with a conventional adjuvant.

There are different methods for entering immune compounds into living organisms’ bodies, at this stage of the study, both through oral and peritoneal injections of recombinant protein OipA and propolis into the body of living organisms so that we can finally obtain a more accurate evaluation of these experiments. These studies also challenge the pathway in which immunogens can function better and more effectively. Since *H. pylori* is a gastrointestinal pathogen, the suggestion was that the entry of the immune agent through the mouth might be more effective. On the other hand, digestive system enzymes could carry out several changes to protein, and propolis exposed them when entering through digestion.

To investigate more precisely, we measured the production of cytokine by the RT-PCR quantitative method. For this purpose, we investigated the expression of these cytokines in spleen tissue. Investigating the secretion of critical cytokines that direct the immune system toward Th1 can indicate the type of immunogenicity that recombinant protein OipA and propolis can cause.

The positive control sample, *H. pylori*-infected mouse, during the experiments was used to investigate the type of immunity and the effect that this bacterium can cause in the colonized state in the stomach of the mouse, which in fact, the type of immunity expressed against this infection when naturally exposed to the bacteria. In the positive control group infected with *H. pylori* pathogenic strain, isolated from patients with gastritis (25, 39), there was a huge increment in IFN-γ, while the rate of IL-4 decreased. This finding can indicate the type of effect this bacterium has on the immune system. Therefore, it could be considered stimulation of the immune system toward the Th1 pathway. Naturally, numerous antigens in live bacteria can cause better and more significant immune responses in the natural pathway of infection.

In general, IFN-γ was a cytokine with significant secretion compared with IL-4 in all groups, but this cytokine showed an increase in secretion in some groups with specific treatments, while IL-4 did not show this increase (Figure 4).

The study of all groups with the negative control group (group treated only with PBS and placed in conditions without bacteria and protein) and positive control showed reflective results (Figure 4).

In the mouse group treated with recombinant OipA protein alone, the amount of IFN-γ in the injectable method was lower than the group of mice treated only by the gavage method, causing an increase in the secretion of IFN-γ cytokine. This difference in immunogenicity stimulation was more specific in two different immunogenicity methods in protein plus propolis. In gavage mode, this increase in IFN-γ expression increased about 3.5 times compared with the negative control, but the emulsion state by oral method caused about 3.4 times the relative increase in cytokine secretion of IFN-γ (Figure 4). These results may indicate the way the antigen enters the body through H. pylori can affect the immune response. In other words, imitation of the natural way through which the cause of infection entered can assume a huge part in choosing the causing immune pathway.

In the propolis treatment group alone, the injectable method was more effective and increased IFN-γ more than oral condition (Figures 4 and 5); thus, it is likely that the entry of propolis through oral instability caused immune conditions, and the optimum concentration that can stimulate immunity in the stomach has likely decreased.

Immunogenicity by Freund’s adjuvant had a much more significant effect on cytokine secretion of IFN-γ than other groups. Of course, in the gavage method, propolis in treatments evaluated as an adjuvant could increase the amount of adjuvant treatment compared with other treatments. This point indicates that propolis can act as a public adjuvant like the Freund’s adjuvant. The fact that IL-4 showed decreasing value in all groups indicates that the immune system might conduct the Th1 pathway.

Comparing the results of other treated groups in which recombinant protein associated with adjuvant indicates the presence of adjuvants, whether propolis or Freund, leads the immune system toward the desired pathway and will be able to modulate the immune response to cellular immunity. Of course, OipA, along with propolis, cannot stimulate more immunogenicity when treated subcutaneously. In contrast, protein and propolis alone provide more immunity through injection, which can be associated with the fact that their combination in injectable mode has inhibitory effects, an inhibitory effect not seen in gavage mode. Perhaps attachment of the recombinant protein to epithelial cells, a feature that this outer membrane protein does in *Helicobacter*, increases immune system irritability. The propolis compound and OipA protein, which are inhibitory on each other in injectable mode, can pass the digesting enzymes in the digestive system and effectively create immunity by reaching the stomach and separating from each other the attached OipA protein. Finally, in injectable mode, the combination of OipA + propolis reduces the immunogenicity that OipA and propolis, alone, can create far more. On the other hand, because propolis alone cannot increase humoral immunity, combining these two does not increase humoral immunity, reducing cellular immunity against OipA, thus, the assumption that propolis by inducing immune inhibitor cytokines reduces OipA immunogenicity. However, investigating the number of other cytokines, such as inhibitory cytokines, will help reject this hypothesis altogether.

In the study conducted by Chen *et al*., 2012, this antigen (OipA protein) effect was evaluated. For this purpose, a DNA construct that encodes the *oipA *gene is used to vaccinate C57BL/6 mice, and after vaccination, *H. pylori* colonization is reduced in mice (40). They found that subunit B encodes the DNA of the heat-labile toxin as an adjuvant modulator of the immune response in mice toward Th1.

In another research, Chen *et al*. (2012) used *Salmonella typhimurium* for *oipA *optimized gene expression for immunization (40). They additionally tracked down that oral *oipA* DNA vaccine in mice, remarkably increased IgG2a/IgG1 antibody and IFN-γ/IL-4 cytokines, indicating a dual immune reaction of Th1/Th2 decreasing the deployment of bacteria in vaccinated mice (41).

Mahboubi *et al*. (2017) have found a similar effect of recombinant protein OipA. They used propolis as adjuvant and recombinant OipA protein as a vaccine antigen. The OipA group and the control group in the C57BL/6 mouse model have shown that the IgA titer was significantly higher. However, they could not see the optimal effect like the propolis adjuvant (25). In this way, the inhibitory effects of using propolis with OipA in mice, which was observed in the Mahboubi *et al*. study, could be due to inappropriate or high concentrations of propolis (25) because by determining the optimum concentration, we were able to prove that propolis, along with the recombinant OipA protein, with gavage method, were synergistic in increasing immunity. At higher concentrations, phenolic compounds, flavonoids, or then again different compounds may bind to OipA and somewhat influence its antigen structure. By choosing a better concentration of OipA and propolis, this possibility may have been prevented, leading to the production of IFN-γ in the animal (26, 41-43) 

Actuation of T lymphocytes causes a progression of falling actions and activities, including enactment of membrane secretion symptoms and expression of cytokine genes—characteristics for example such as solidity and synthesis pace of mRNA and protein change. This process results in the expansion and differentiation of T cells and production of cytokines (44). A fundamental functional feature of immune system cells is the capacity to synthesize and discharge cytokines that tight spot to explicit receptors on the outside of an objective cell. Once connected, cytokines go about as growth regulators or differentiation of these cells and improve the immune response (45). The creation of a cellular or humoral immune response relies on an extensive range of cytokines secreted by different cells, including TTCD4+ (Th1 and Th2) TCD8+. IFN-γ secreted by Th1 cells is a basic cytokine in the cellular immune response ordinarily known as a defense mechanism against viral contamination (46). A study showed that in mice, sublingual (SL) vaccination could adequately prompt protection against *H. pylori* infection in collaboration with vigorous T and B cell invasion into the stomach (47). Researchers examined the likely role of Urease subunit B (UreB) in the induction of Th17 cell response and proposed that UreB can get Th17 cell reactions against *H. pylori* both *in vivo* and *in vitro* (48).

In addition to increasing the strength of the humoral immune response, propolis extract also allows for increased cellular response and increases mRNA synthesis from IFN-γ. The expression of IFN-γ in mice splenocytes immunized with recombinant OipA protein plus immunized propolis was higher, indicating a higher level of protected animals in this group after the test. These outcomes resembled those obtained by Blonska *et al*. (2004), who had worked with a European propolis sample and had acted on ethanol extract to regulate gene expression at the transcription level (49). Other researchers have additionally reported an expansion in cytokine discharge in mice getting propolis (50) Using Brazilian or European propolis. A study (2003) detailed that propolis has a regulatory impact straightforwardly on the essential useful qualities of the immune system, by the ErK2 map-kinase mark which is engaged in the procedure that enhances cellular growth (50). However, it must be emphasized that due to high discrepancies in the chemical compositions of propolis in different regions, it is essential to analyze the chemical compositions and examine the quality control process before utilization of propolis as an adjuvant.

In another study, a multilateral analysis of ethanol concentrates of various samples to determine the degree of bioactive substances by high-performance liquid chromatography (HPLC) empowered the typing of Brazilian propolis (27). In addition to high amounts of phenolic compounds such as artepillin-C, green propolis has cinnamic acid and flavonoids such as pinobanksin and kaempferol. Of course, the specific component of propolis activity is obscure (50); nonetheless, these substances may stimulate immune system cells to secret cytokines such as IL-1, IL-6, IL-8, IL-12, and IFN-γ, which can stimulate humoral immunity just as a cellular immune response (51). 

The present study showed that recombinant OipA protein could stimulate the immune response related to Th1 by increasing the critical cytokine IFN-γ.

It also observed that the ethanolic extract of propolis goes about as a modulator of the immune system. In OipA-immunized mice, adjuvant propolis enlarged the strength of the cellular immune response, leading to increased protection. Thus, propolis as an adjuvant may help the effectiveness of vaccines, especially in cases requiring a cellular immune reaction for a protective reaction.

Another critical point was that apparently, the sum of recombinant protein and adjuvant could create appropriate immunogenicity, which of course, the way the immune system induced in this study looks more effective. However, individually, this effect was much less in increasing IFN-γ expression for both propolis and recombinant OipA protein.

In this experiment, as we have seen, two oral and injecting methods of antigen entries were also investigated, but it seems that oral entry can stimulate the cellular immune system in larger quantities by recombinant OipA protein.

The fact that IFN-γ increased in all treated groups but IL-4 decreased or did not change strengthens the theory that this protein and propolis may enhance cellular immunity. Therefore, the obtained results can prove the immunogenicity of recombinant OipA protein, which can be used in later stages of research to design a suitable vaccine. Although OipA stimulates cellular immunity better than other pathogens of *H. pylori*, it is necessary to investigate the level of IgG and IgA antibody titers in serum. Further large-scale studies are needed to evaluate the immunogenicity of the designed recombinant OipA protein. In addition, *H. pylori* deployment evaluation after treating mice with this protein will help see the prophylaxis or preventive effect of this vaccine.

On the other hand, despite stimulating cell immunity by this protein, it is better to investigate the possible cellular immune effects on the gastric tissue by examining the impressions of cellular immunity on the tissue itself to see the devastating effects that may be caused and lead to necrosis or excessive inflammation in the gastric tissue.

## Conclusion

 The results confirm the efficiency of OipA in the induction of IFN-γ production, thereby the cellular immune response. The role of propolis as a suitable adjuvant is suggested. The result of this study also highlights the role of vaccine therapies in the better management of infections.

## Authors’ Contributions

HS and TF designed the study and wrote the manuscript, also provided vital analytical tools. HS performed the majority of experiments. SG and MM participated in analytical tools and critically read the manuscript.

## Conflicts of Interest

The results presented in this paper were part of a student thesis. The authors have no financial or commercial conflicts of interest with the current work or its publication.
